# Malaria and anaemia prevalence and associated factors among pregnant women initiating antenatal care in two regions in Ghana: an analytical cross-sectional study

**DOI:** 10.1186/s12884-025-07735-5

**Published:** 2025-05-27

**Authors:** Gifty Dufie Ampofo, Joseph Osarfo, Doris Dokua Okyere, Ekoue Kouevidjin, Matilda Aberese-Ako, Harry Tagbor

**Affiliations:** 1 University of Health and Allied Sciences, Ho, Ghana; 2 Université Cheikh Anta Diop, Dakar, Senegal

**Keywords:** Malaria parasite infection, Malaria in pregnancy, Anaemia in pregnancy, Prevalence, Risk factors, Antenatal care, Ghana

## Abstract

**Background:**

Malaria and anaemia in pregnancy remain public health problems because they increase the risk of adverse pregnancy outcomes. This study assessed malaria and anaemia prevalence and associated risk factors among pregnant women initiating antenatal care in selected districts of 2 regions of Ghana.

**Methods:**

An analytical cross-sectional study was conducted using data obtained from 5196 pregnant women at their booking antenatal care (ANC) visit. Women of any age, gestational age, parity and at any ANC visit, who consented were enrolled consecutively into the study. Data on socio-demographic and obstetric characteristics, bed net ownership and use were obtained using structured questionnaires. Haemoglobin concentration and malaria, Schistosoma and helminth infections were determined using an automated haematology analyser and microscopy, respectively. Summary statistics to describe study variables and chi-square test and logistic regression set at *p* < 0.05 to determine risk factors for anaemia and malaria were conducted using Stata SE14.

**Results:**

Overall malaria prevalence was 5.74% [95% CI: 5.1–6.4] and anaemia prevalence was 55.22% [ 95% CI: 53.85–56.58]. Living in Volta region (*p* < 0.001), being secondi- (*p* = 0.003) or multigravida (*p* < 0.001) and being of lower middle socio-economic status (*p* = 0.004) reduced the women’s risk of malaria parasite infection. Being anaemic (*p* = 0.001) and reporting a symptom (*p* < 0.001) increased the odds of Plasmodium infection among the women. Residing in Volta region (*p* < 0.001), having malaria infection (*p* < 0.001), and booking ANC in the 2nd (*p* < 0.001) and 3rd trimesters (*p* < 0.001) increased the odds of anaemia among the women. Age 25–34 years (*p* < 0.001) and ≥ 35 years (*p* = 0.008) and belonging to middle (*p* = 0.009), upper middle (*p* = 0.006) or upper-level (*p* < 0.001) quintile of wealth index reduced the odds of anaemia among the women at their booking ANC visit.

**Conclusions:**

More than half the women were anaemic signifying a severe public health problem. Malaria prevalence, though low, was a significant risk factor for anaemia. Existing malaria and anaemia control strategies through ANC need strengthening, especially among young, first-time pregnant women. This study further highlights socio-economic status as an important risk factor for anaemia in pregnancy.

**Trial registration:**

Not applicable.

**Supplementary Information:**

The online version contains supplementary material available at 10.1186/s12884-025-07735-5.

## Background

Malaria and anaemia in pregnancy (Haemoglobin concentration (Hb) < 11 g/dl) remain public health problems, because they affect significant numbers of populations, especially in sub-Saharan Africa [1, 2]. They increase the risk of prenatal, perinatal, neonatal and maternal morbidity and mortality [3, 4]. Malaria in pregnancy (MiP) increases the risk of maternal anaemia, foetal growth restrictions, low birth weight (LBW), preterm deliveries, miscarriages, stillbirths and sometimes maternal death [3, 5–7]. Likewise, anaemia in pregnancy (AiP) is associated with LBW, small-for-gestational age babies, preterm deliveries, perinatal and neonatal mortality, post-partum haemorrhage and maternal death [4, 8–10]. In 2018 alone, it was estimated that 11 million out of 38 million pregnancies in sub-Saharan Africa (sSA) were exposed to malaria infection which led to an estimated 872, 000 (MiP) related LBW babies [1]. Similarly, in 2021 and 2022, 13.3 million and 12.7 million out of estimates of 40 million and 35.4 million pregnancies respectively in the WHO African region were exposed to malaria infection [11, 12]. These exposures to malaria infection would have resulted in an estimated 961, 000 and 914, 000 babies born LBW in 2021 and 2022 respectively if no intervention was implemented [11, 12]. The overall prevalence of asymptomatic malaria infection among pregnant women in sSA between 2002 and 2020 has been estimated to be 26.1%, with *Plasmodium falciparum* being the dominant species (98.6%) [13]. An estimated 15% of babies were born LBW in Africa [14] while a pooled-prevalence of 9.76% LBW has been estimated in 35 sSA countries using demographic health surveys (DHS) conducted between 2008 and 2018 [15].

Global estimates of AiP prevalence stand at 36.8% [16], however, in sSA estimates of 50% have been reported from 26 countries using DHS conducted between 2010 and 2019 [17]. In a systematic review, an estimated 42.7% of pregnant women were anaemic in lower- and middle-income countries between the late 1990’s and 2015 [4] compared to only 12.8% between 2004 and 2016 in a developed country [10].

Among the complex interplay of multiple known risk factors of maternal anaemia, malaria infection seems key [18–22]. In a recent systematic review, pregnant women with asymptomatic malaria infection had 2.28 times higher odds of anaemia compared to pregnant women not infected [13]. Other known risk factors for AiP include other infections like helminthiasis, schistosomiasis, Human Immuno deficiency Virus (HIV) and Tuberculosis [23–27]. Additionally, poor maternal nutrition including nutritional deficiencies, especially iron deficiency and food insecurity have been reported [28, 29]. Sociodemographic and behavioural factors like younger age, lower socio-economic status, being single, lower educational level, rural residence, primi and multi gravidity and parity, poor antenatal care (ANC) seeking behaviour, lack of health insurance and late ANC initiation are also known risk factors for AiP [28–30].

World Health Organisation (WHO)-recommended control strategies for MiP and AiP in malaria endemic countries within the context of ANC include the delivery and encouraging the use of insecticide treated bed nets (ITN), the administration of intermittent preventive treatment of malaria in pregnancy using sulfadoxine-pyrimethamine (IPTp-SP), prompt and effective case management of malaria, daily iron and folic acid supplementation (IFAS), preventive anti-helminthic treatment, prevention of mother-to-child transmission of HIV and syphilis and giving of dietary advice [5, 31]. In Ghana, these control interventions have been implemented through the routine ANC system for over two decades. Although implementation challenges have resulted in lower-than-expected coverages [32–35], there has been gradual improvement in their uptake over the years [36]. For example, ITN use among pregnant women increased from 3% in 2003 to 49% in 2019 and pregnant women receiving 3 or more doses of IPTp-SP increased from 27% in 2008 to 61% in 2019 [37–39]. There is also evidence of a decline in MiP prevalence across the country [40]. In the 1990’s, reports of MiP prevalence as high as 60% and above were documented in the northern savannah and middle transitional zones of the country [41, 42]. These reduced by more than 50% between 2014 and 2017 [43–45]. Similarly, in the coastal savannah zone, a decline from almost 20% MiP prevalence in the early 2000’s to 10.1% in 2018 has been reported [46, 47]. However, the continued improvement in uptake of ANC control interventions coupled with decreasing MiP infection prevalence, has not resulted in a commensurate decline of maternal anaemia [22, 36]. AiP still remains a moderate to severe public health problem across the country. National trends using the Ghana’s District Health Information Management System (DHIMS) 2 data reported increasing AiP prevalence at booking ANC visit from 31 to 36.7% between 2012 and 2018 [36]. Higher AiP prevalence at booking ANC visit between 44.1% and 66.4% have also been reported in epidemiological studies conducted between 2012 and 2018 [43, 48–52].

Whilst Ghana seems to be making strides with reducing the malaria infection burden among pregnant women, it cannot boast of reaching the WHO Global nutrition targets of 50% reduction of anaemia in women of reproductive age over the 2012 figure by 2025 [53]. Studies to continuously assess the burden of both malaria and anaemia in pregnant women are essential to monitor progress of their control. This study aimed at assessing the prevalence of malaria parasite infection and anaemia and associated factors among pregnant women at their first ANC visits in selected districts of 2 regions of Ghana. Findings from the study will establish the prevailing burden of MiP and AiP. Risk factors of MiP and AiP that need to be emphasised when implementing control interventions will also be highlighted. These will inform targeted strategies aimed at the control and subsequent elimination of malaria and anaemia in pregnancy in the country.

## Methods

### Study design and population

This study was an analytical cross-sectional study using data from a cohort of pregnant women at their booking ANC visit, enrolled into a health facility-based non interventional study that aimed at determining the public health significance of factors contributing to maternal anaemia at term pregnancy and LBW. It was conducted in the Ashanti and Volta regions of Ghana. Pregnant women of any age, parity and gestational age, at any point of their ANC and willing to comply with ANC schedules throughout their pregnancy were enrolled into the cohort. Women who were ill or needed hospitalization were excluded. Pregnant women who were not recruited at their first ANC visit had their booking ANC information and laboratory investigation results recorded retrospectively from their maternal and child health record books (MCHRB). The pregnant women were sampled consecutively in each of the ANC clinics. Enrolment was simultaneously done in all the selected health facilities during the same period. The numbers enrolled per each facility were pooled together on a weekly basis until the estimated number of women for the main study was attained.

### Study area

**Fig. 1:**
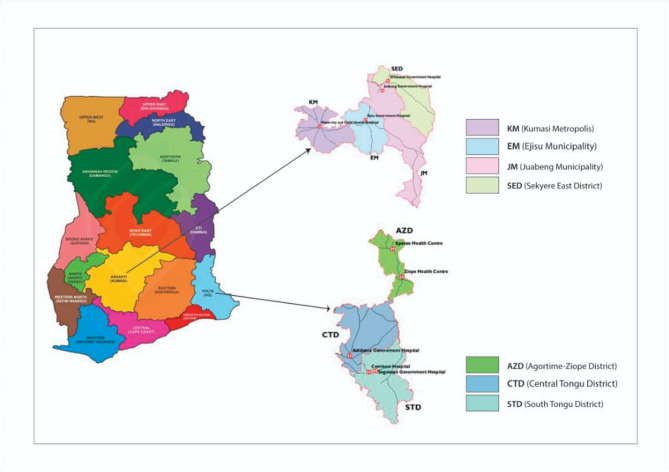
Map of Ghana showing the study sites in the Ashanti and Volta Regions

The study was conducted in selected districts in the Ashanti and Volta Regions of Ghana (Fig. 1). The Ashanti region lies in the middle belt of the country characterised by transitional savannah and forest vegetation with perennial and moderately intense malaria transmission while the Volta Region spans across both the transitional forest zone and the coastal savannah zone along the coast of the Atlantic Ocean with a less intense transmission of malaria [54]. The main economic activity carried out in both regions is subsistence farming.

Three administrative areas in the Ashanti Region: Sekyere-East District (SED), Ejisu-Juaben Municipality (EJM) [now separated into the Ejisu Municipality (EM) and the Juaben Municipality (JM)] and Kumasi Metropolis (KM) and 2 in the Volta region of Ghana: Agortime-Ziope District (AZD) and South Tongu District (STD) were included (Fig. 1). These areas were purposively selected for the study to be easily accessible and include urban, semi-urban and rural areas. The SED is mainly rural, EJM semi-urban and KM mainly urban while AZD is mainly rural and STD is largely urban. The semi-urban area in the Volta region (Central Tongu District-CTD) was excluded because there was a similar study ongoing at the only district hospital and the clinics available had very few antenatal care attendances.

In all, 4 health facilities in each region were included in the study: 4 hospitals in Ashanti and 2 hospitals and 2 health centres in Volta. The hospital in KM although located in an urban area also receives rural dwellers for their ANC services as it is situated close to the central market that attracts them for trading of food stuffs. AZD has no hospitals and hence the two health centres situated there were included. All health facilities carry out antenatal care and delivery services and attend to at least 20 ANC registrants (booking attendants) per week. In 2017, the ANC coverage, proportion of ANC registrants receiving an ITN and the prevalence of anaemia at first ANC visit were 73.8%, 55.9% and 30.1% respectively in the Ashanti region compared to 60.5%, 95.5% and 35.5% respectively in the Volta region [55].

### Sample size estimation

Malaria infection during pregnancy is key among the multiple risk factors associated with maternal anaemia and low birth weight. Using a malaria prevalence of 11.0% [43, 56] an estimated minimum sample size of 5000 pregnant women (for maternal anaemia) and 2500 live births (for LBW) were deemed sufficient to give the main study at least 80% power to detect a Population Attributable Fraction (PAF-the proportion of maternal anaemia and LBW in the study population that can be attributed to malaria infection in pregnancy) of at least 10% (public health significance) using methods described by Browner and Newman [57].

### Study procedures at enrolment of study participants

At each of the selected ANC clinics, the pregnant women received general information about the study at the beginning of each ANC session from a designated ANC staff. After screening, eligible women were given further information about the study by trained Research Assistants regarding the purpose, risks and benefits, confidentiality, voluntariness, and compensation for their participation. Their written informed consent was obtained either by signing or thumbprinting for those who could not write when they agreed to participate, and they were then enrolled. Pregnant women < 18 years were assented while consent for their participation was obtained from their legally acceptable representatives who were adults accompanying them for their ANC. The women were afterwards administered an electronic-based structured questionnaire by the Research Assistants to collect data on several individual and community level variables that have been theoretically or empirically linked to pregnancy and birth outcomes. These included their socio-demographic and socio-economic characteristics, obstetric history, any presenting complaints/symptoms and ITN ownership and use. After their initial consultations with the ANC staff, additional information on any presenting complaints, measurements of height, weight, blood pressure and symphysio-fundal height and test results for HIV, syphilis and hepatitis B screening (which were conducted at the ANC using rapid diagnostic tests) were recorded from their MCHRB. The women were then asked to visit the laboratories for their ANC laboratory investigations where study samples were also taken. After their return from the laboratories to the consulting rooms, blood tests results including Glucose-6-Phosphate Dehydrogenase (G6PD) deficiency - and sickle cell statuses were recorded from their MCHRB. The study procedures were piloted at each study site on three consecutive days before the official start of enrolment of study participants. The results from the pilot study were not included in the final analysis.

### Study samples collection

Two (2) mls venous blood was obtained by trained laboratory staff from each study participant into a K2-Ethylenediamine tetra acetic acid tube for full blood count (FBC) assessment, and preparation of thick and thin blood smears for determination of plasmodium parasite using microscopy. Blood samples were stored in refrigerators at 2–8 ^0^C at the health facility’s laboratories (for a maximum of 7 h) until the end of each ANC session when they were transported to central laboratories (Juaben Government Hospital in Ashanti and Ho Teaching Hospital in the Volta Regions) in cold boxes for FBC and malaria microscopy. Study participants were also given well-labelled urine and stool containers and coached to present early morning mid-stream urine catches and thumb-sized stool samples at their next ANC visits for urine and stool examinations respectively. Upon receiving stool samples from the pregnant women, they were initially processed by emulsifying the sample in 4 ml of 10% formalin in the stool container and stored at 2-8^0^ C at the health facilities’ laboratories. Urine samples were refrigerated immediately without any processing. Both samples were transported in cold boxes to the central laboratories at the end of the ANC session for analysis.

### Measurement of hb

Hb measurement was carried out using SYXMEX KX-21 haematology analyzer (Sysmex Corporation Kobe, Japan) as part of a full blood count. The analysers used underwent regular routine maintenance and servicing and daily quality control using control samples to assure high quality of the results.

### Microscopy for malaria parasite determination

Thick and thin blood smears were air-dried, thin smears fixed in absolute methanol and both Giemsa stained for evaluation of malaria parasitaemia, asexual forms and gametocytes using microscopy. Parasite densities were computed from thick films as the number of asexual parasites per 200 leukocytes (or per 500 leukocytes, if the count was < 10 asexual parasites/200 leukocytes), assuming a leukocyte count of 8,000/µl [58]. A blood smear was considered negative when the examination of 100 high power fields did not reveal asexual parasites or gametocytes. Thin blood smears were evaluated for parasite species. For quality control, all slides were double read by two independent microscopists. Both microscopists were certified medical laboratory scientists who underwent a standardized pre-study training and assessment. If there was any discrepancy in the reading of the slides, a third certified laboratory scientist was involved in reading the slide to resolve the discrepancy. In addition, more than 10% of all slides from each study site were sent for review by a WHO accredited microscopist at the Kumasi Centre for Collaborative Research in Ghana.

### Urine and stool examination

Urine specimens were examined the same day of collection to identify ova of *Schistosoma haematobium* using the centrifugation sedimentation technique [59]. After 5 min of centrifuging the urine sample, and decanting the supernatant, a drop of well-mixed sediment was examined under the microscope to detect any parasitic ova. Stools were also examined using the formalin ethyl-acetate concentration technique for microscopic detection of ova of intestinal helminths [60].

### Data management

Data were collected by trained Research Assistants using personal digital assistants. The personal digital assistants were programmed with an electronic data capture system developed in xlsform (xlsform.org) for use with SurveyCTO (Dobility, Inc; Cambridge, MA, USA). The electronic forms were designed from initial paper-based questionnaires and piloted in the field prior to use. Completed forms on personal digital assistants were uploaded by the Research Assistants on to the SurveyCTO platform in real time after site supervisors checked for accuracy and completeness. Data collected at each ANC facility were maintained in a harmonized central database with site specific and central quality control procedures. In addition, data checks for key variables were run on data downloaded periodically from the SurveyCTO platform by the Data manager. Pooled, de-identified data from all sites were maintained on a central, password- protected SurveyCTO server accessible to only the Data manager and the project PI.

### Data analysis

Data were analysed using Stata version 16 (Stata, College Station, TX, USA). Data were summarised as proportions and frequencies for categorical variables. Means with their respective standard deviations were used to summarise data if the variables were continuous and normally distributed. Chi-square tests were used to compare proportions while Students’ t-test to were used to compare means of covariates. Bivariate analyses were used to test the independent associations between each covariate and the outcome variables (maternal anaemia and malaria parasite infection at booking). Correlations among all covariates were tested with the Pearson’s R statistic to identify collinear pairs. Only those variables which were statistically significant at *p* < 0.1 and non-collinear (Pearson correlation *r* < 0.5) variables associated with maternal anaemia and malaria parasite infection were advanced to build backward fitting logistic regression models; first removing variables that were least associated with maternal anaemia and malaria parasite infection and retaining those variables that were associated with maternal anaemia and malaria parasite infection at booking (*p* < 0.05).

### Description of variables

Principal components analysis was used to derive a wealth index variable of the pregnant women, based on each woman’s educational level, occupation, marital status, housing characteristics and ownership of assets. The housing characteristics included the type of floor, roof, toilet facility, electricity, water and fuel for cooking. The assets included ownership of radio, television, mobile or fixed phone, refrigerator, car, bicycle or motorcycle and ITN. The wealth index was categorized into five quintiles: lower, lower middle, middle, upper middle and upper [61].

Based on WHO definitions, anaemia was defined as Hb < 11.0 g/dL. Mild, moderate and severe anaemia were Hb levels of 10.0-10.9, 7.0-9.9 and < 7.0 g/dL respectively [62]. Primigravida, secundigravidae or multigravidae referred to women who had been pregnant only once, twice or more than twice respectively, including the index pregnancy that the woman was reporting to the ANC clinic with. The gestational age at recruitment was categorized into first (1 to 12 weeks), second (13 to 24 weeks) and third (25 weeks and above) trimesters.

### Ethical considerations

Ethical approval for the study was obtained from the University of Health and Allied Sciences Research Ethics Committee (Certificate number: UHAS-REC/A.1 [l] 17–18). Permission was also sought from the district directors of health services and the heads of the health facilities and ANC clinics that were included in the study. Written informed consent was obtained from all pregnant women who participated in the study. Pregnant women who were below the age of 18 years were assented to participate in the study while consent was sought from their guardians who accompanied them to the ANC clinic. Coded study identification numbers rather than names were used to identify the participants during electronic data capture. The risks associated with participation were minimal, essentially no greater than during routine antenatal care in Ghana which included pricks for drawing venous blood and extra time spent for data collection. Certified laboratory scientists and well-trained Research Assistants were involved with collection of samples and data to minimize any potential harm to study participants. The Research Assistants were either community health nurses or undergraduates with a health background. Participants were compensated with bars of soap at recruitment and a delivery package of disinfectant, a bar of soap and a face towel at delivery.

## Results

### Socio-demographic characteristics

Women were enrolled simultaneously at all the ANC clinics, in both regions, from May 2018 to March 2020. Table 1 below summarises the women’s socio-demographic characteristics. A total of 5196 pregnant women were enrolled: 2333 (44.90%) and 2863 (55.10%) from the Ashanti and Volta regions respectively.

**Table 1:** Socio-demographic characteristics of study participants

Measured parameters	Number of participants	Percentage of participants (95%CI)
**Region**		
Ashanti	2333	44.90 (43.55–46.26)
Volta	2863	55.10 (53.74–56.45)
**Age (years)**		
< 25	1839	35.69 (34.39–37.01)
25–34	2510	48.71 (47.35–50.08)
≥ 35	804	15.60 (14.64–16.62)
**Gravidity**		
Primigravidae	1188	23.11 (21.98–24.28)
Secundigravidae	1131	22.00 (20.89–23.15)
Multigravidae	2822	54.89 (53.53–56.25)
**Gestational age (weeks)**		
First trimester	2295	46.60 (45.21–47.99)
Second trimester	2114	40.51 (39.14–41.89)
Third trimester	516	12.89 (11.99–13.86)
**Highest educational level**		
None	380	7.39 (6.71–8.14)
Primary	823	16.01 (15.03–17.04)
Junior High School	2477	48.18 (46.82–49.55)
Senior High School	983	19.12 (18.07–20.22)
Tertiary	478	9.30 (8.53–10.12)
**Wealth index**		
Lower	1029	20.02 (18.94–21.13)
Lower middle	1028	20.00 (18.92–21.11)
Middle	1028	20.00 (18.92–21.11)
Upper middle	1028	20.00 (18.92–21.11)
Upper	1028	20.00 (18.92–21.11)
**Marital status**		
Not married	1677	32.62 (31.35–33.91)
Married	3464	67.38 (66.09–68.65)
**Own an ITN**		
No	721	19.19 (17.96–20.48)
Yes	3037	80.81 (79.52–82.04)
**Slept under ITN night prior to enrolment**		
No	1509	40.15 (38.60–41.73)
Yes	2249	59.85 (58.27–61.40)
**Employment status**		
Unemployed	773	15.00 (14.05–16.01)
Employed	979	19.00 (17.95–20.10)
Self employed	3400	65.99 (64.69–67.28)

The mean (SD) age of the women was 27.30 (6.50) years. The mean gestational age at booking was 15.50 (8.37) weeks. More than half (54.89%) of the women were multigravidae and close to half of them (46.60%) had their booking ANC visit in the first trimester. More than 90% (92.61%) of the women have had at least primary level education, more than two-thirds (67.38%) were married and 84.99.% were employed. An equal number of 20.00% of the women belonged to each of the quintiles of the wealth index. Overall, 80.81% of the women reported owning an insecticide treated net but only 59.85% of them slept under an ITN the night prior to enrolment.

Headache, lower abdominal pain, loss of appetite and dizziness constituted majority (62.40%) of the clinical symptoms reported at booking ANC visit (Additional file 1: Table S1).

### Key laboratory investigations at booking ANC visit

Overall, the prevalence of malaria parasite infection at booking was 5.74% [95% CI: 5.11–6.44] (Table 2) and the geometric mean parasite density (GMPD) was 2609 parasites per microlitre. *Plasmodium falciparum* constituted 94.30% of the parasites detected, *P. malariae* 5.20% and *P.ovale* 0.50%. No *P. vivax* was detected. The prevalence of malaria parasite infection in the Ashanti and Volta regions was 10.24% [95% CI: 8.92–11.68] and 2.63% [CI: 2.07–3.29] respectively (Refer to Table 3 for percentages). The GMPD in the Ashanti region was 982 parasites per microlitre compared to 18,226 parasites per microlitre in the Volta region.

**Table 2:** Key laboratory investigation results of women at booking ANC visit

Measured parameters	Number of participants	Percentage of participants (95% CI)
**Malaria parasitaemia**		
No	4432	94.26 (93.56–94.89)
Yes	270	5.74 (5.11–6.44)
**Hb category**		
Severe anaemia (Hb < 7 g/dl)	77	1.49 (1.2–1.86)
Moderate anaemia (Hb = 7.0–9.9 g/dl)	1344	26.07 (24.89–27.28)
Mild Anaemia (Hb = 10.0–10.9 g/dl)	1426	27.66 (26.45–28.89)
No Anaemia (Hb ≥ 11.0 g/dl)	2309	44.78 (43.43–46.14)
**HIV status**		
Negative	4329	98.54 (98.14–98.86)
Positive	64	1.46 (1.14–1.86)
**Syphilis status**		
Negative	2732	97.26 (96.59–97.8)
Positive	77	2.74 (2.20–3.41)
**Hepatitis B surface antigen**		
Negative	3353	94.42 (93.62–95.13)
Positive	198	5.58 (4.87–6.38)
**G6PD status**		
Negative	1598	94.95 (93.79–95.90)
Positive	85	5.05 (4.10–6.21)
**Hb sickling status**		
Negative	2805	88.85 (87.7–89.90)
Positive	352	11.15 (10.10–12.30)
***S. haematobium***		
Negative	4652	99.64 (99.42–99.79)
Positive	17	0.36 (0.21–0.58)

**Table 3:** Factors associated with malaria parasite infection at booking

	Malaria parasitaemia (%)		Unadjusted OR (95% CI)	*p*-value	Adjusted OR (95% CI)	*p*-value
	Negative	Positive				
**Region**						
Ashanti	1727 (38.97)	197 (72.96)	Reference		Reference	
Volta	2705 (61.03)	73 (27.04)	0.24 (0.18–0.31)	< 0.001	0.14 (0.09–0.21)	< 0.001
**Anaemia**						
No	1980 (44.94)	89 (32.96)	Reference		Reference	
Yes	2426 (55.06)	181 (67.04)	1.66 (1.28–2.15)	< 0.001	1.83 (1.29–2.59)	0.001
**Age (years)**						
< 25	1548 (35.09)	146 (54.28)	Reference		Reference	
25–34	2168 (49.15)	92 (34.20)	0.45 (0.34–0.59)	< 0.001	0.75 (0.48–1.17)	0.204
≥ 35	695 (15.76)	31 (11.52)	0.47 (0.32–0.70)	< 0.001	0.93 (0.51–1.71)	0.823
**Gravidity**						
Primigravidae	969 (22.02)	106 (39.41)	Reference		Reference	
Secundigravidae	964 (21.91)	61 (22.68)	0.58 (0.42–0.80)	0.001	0.50 (0.32–0.79)	0.003
Multigravidae	2467 (56.07)	102 (37.92)	0.38 (0.29–0.50)	< 0.001	0.31 (0.19–0.50)	< 0.001
**Trimester at booking**						
First	2055 (48.85)	82 (32.16)	Reference		Reference	
Second	1756 (41.74)	139 (54.51)	1.98 (1.50–2.63)	< 0.001	1.10 (0.77–1.58)	0.595
Third	396 (9.41)	34 (13.33)	2.15 (1.42–3.26)	< 0.001	1.22 (0.73–2.05)	0.453
**Highest educational level**						
None	321 (7.30)	28 (10.41)	Reference		Reference	
Primary	741 (16.84)	37 (13.75)	0.57 (0.34–0.95)	0.031	0.95 (0.45–2.02)	0.891
JHS	2134 (48.50)	139 (51.67)	0.75 (0.49–1.14)	0.176	1.19 (0.61–2.34)	0.614
SHS	785 (17.84)	56 (20.82)	0.82 (0.51–1.31)	0.404	0.90 (0.41–1.96)	0.784
Tertiary	419 (9.52)	9 (3.35)	0.25 (0.11–0.53)	< 0.001	0.37 (0.13–1.06)	0.064
**Wealth index**						
Lower	925 (21.02)	68 (25.28)	Reference		Reference	
Lower middle	928 (21.09)	55 (20.45)	0.81 (0.56–1.16)	0.250	0.48 (0.29–0.80)	0.004
Middle	917 (20.84)	59 (21.93)	0.88 (0.61–1.26)	0.469	0.75 (0.46–1.22)	0.251
Upper middle	888 (20.18)	49 (18.22)	0.75 (0.51–1.10)	0.138	0.58 (0.33–1.03)	0.062
Upper	742 (16.86)	38 (14.13)	0.70 (0.46–1.05)	0.083	0.80 (0.41–1.55)	0.500
**Own an ITN**						
No	612 (18.44)	40 (20.83)	Reference		Reference	
Yes	2707 (81.56)	152 (79.17)	0.86 (0.6–1.23)	0.407	1.11 (0.79–1.56)	0.556
**Slept under ITN night prior to enrolment (ITN use)**						
No	1279 (38.54)	79 (41.15)	Reference		Reference	
Yes	2040 (61.46)	113 (58.85)	0.9 (0.67–1.21)	0.470	1.13 (0.75–1.70)	0.555
**Report of symptoms at booking**						
No symptom	2307 (52.28)	129 (47.96)	Reference		Reference	
At least one symptom	2106 (47.72)	140 (52.04)	1.19 (0.93–1.52)	0.169	1.82 (1.31–2.52)	< 0.001
**Employment status**						
Unemployed	705 (15.99)	29 (10.78)	Reference		Reference	
Employed	889 (20.16)	37 (13.75)	1.01 (0.62–1.66)	0.963	1.68 (0.89–3.16)	0.108
Self employed	2815 (63.85)	203 (75.46)	1.75 (1.18–2.61)	0.006	1.46 (0.83–2.56)	0.193

More than half (55.22% [ 95% CI: 53.85–56.58]) of the women had anaemia (Hb < 11 g/dL); 1.49% being severely anaemic (Hb < 7 g/dl). The mean (SD) of the Hb was 10.7 (1.5) g/dl. Maternal anaemia was more common among women enrolled in the Volta region (65.56% [95% CI: 63.78–67.31]) than among women in the Ashanti region (42.56% [95% CI: 40.53–44.60]) (Refer to Table 4 for percentages).

**Table 4:** Factors associated with maternal anaemia at booking

	Maternal Anaemia (%)		Unadjusted OR (95% CI)	*p*-value	Adjusted OR (95% CI)	*p*-value
	No	Yes				
**Region**						
Ashanti	1332 (57.69)	987 (34.67)	Reference		Reference	
Volta	977 (42.31)	1860 (65.33)	2.57 (2.29–2.88)	< 0.001	3.21 (2.59–3.98)	< 0.001
**Malaria**						
No	1980 (95.7)	2426 (93.06)	Reference			
Yes	89 (4.30)	181 (6.94)	1.66 (1.28–2.15)	< 0.001	2.09 (1.36–3.22)	< 0.001
**S. Haematobium**						
No	1768 (99.83)	2306 (99.4)	Reference		Reference	
Yes	3 (0.17)	14 (0.60)	3.58 (1.03–12.47)	0.045	2.05 (0.45–9.37)	0.356
**Age (years)**						
< 25	635 (27.74)	1186 (41.92)	Reference		Reference	
25–34	1281 (55.96)	1215 (42.95)	0.51 (0.45–0.58)	< 0.001	0.57 (0.45–0.72)	< 0.001
≥ 35	373 (16.30)	428 (15.13)	0.61 (0.52–0.73)	< 0.001	0.66 (0.49–0.90)	0.008
**Gravidity**						
Primigravidae	459 (20.1)	721 (25.54)	Reference		Reference	
Secundigravidae	515 (22.55)	609 (21.57)	0.75 (0.64–0.89)	0.001	1.13 (0.87–1.47)	0.345
Multigravidae	1310 (57.36)	1493 (52.89)	0.73 (0.63–0.83)	< 0.001	1.29 (0.99–1.68)	0.062
**Trimester at booking**						
First	1239 (56.14)	1040 (38.72)	Reference		Reference	
Second	785 (35.57)	1318 (49.07)	2.00 (1.77–2.26)	< 0.001	1.86 (1.57–2.21)	< 0.001
Third	183 (8.29)	328 (12.21)	2.14 (1.75–2.60)	< 0.001	2.39 (1.68–3.40)	< 0.001
**Highest educational level**						
None	157 (6.87)	217 (7.69)	Reference		Reference	
Primary	272 (11.91)	545 (19.31)	1.45 (1.13–1.86)	0.004	1.22 (0.86–1.73)	0.273
JHS	1075 (47.07)	1389 (49.20)	0.93 (0.75–1.17)	0.549	1.11 (0.80–1.55)	0.522
SHS	495 (21.67)	482 (17.07)	0.70 (0.55–0.90)	0.004	1.00 (0.67–1.48)	0.999
Tertiary	285 (12.48)	190 (6.73)	0.48 (0.37–0.64)	< 0.001	1.42 (0.86–2.35)	0.167
**Wealth index**						
Lower	305 (13.35)	718 (25.43)	Reference		Reference	
Lower middle	358 (15.67)	662 (23.45)	0.79 (0.65–0.95)	0.011	0.84 (0.66–1.07)	0.159
Middle	446 (19.53)	576 (20.40)	0.55 (0.46–0.66)	< 0.001	0.71 (0.55–0.92)	0.009
Upper middle	543 (23.77)	476 (16.86)	0.37 (0.31–0.45)	< 0.001	0.66 (0.50–0.89)	0.006
Upper	632 (27.67)	391 (13.85)	0.26 (0.22–0.32)	< 0.001	0.44 (0.29–0.65)	< 0.001
**Own an ITN**						
No	296 (18.78)	419 (19.43)	Reference		Reference	
Yes	1280 (81.22)	1738 (80.57)	0.96 (0.81–1.13)	0.622	0.87 (0.70–1.08)	0.202
**Slept under ITN night prior to enrolment (ITN use)**						
No	717 (45.49)	784 (36.35)	Reference		Reference	
Yes	859 (54.51)	1373 (63.65)	1.46 (1.28–1.67)	< 0.001	1.1 (0.92–1.31)	0.314
**Report of symptoms at booking**						
No symptom	1282 (55.96)	1509 (53.34)	Reference		Reference	
At least one symptom	1009 (44.04)	1320 (46.66)	1.11 (0.99–1.24)	0.061	1.02 (0.86–1.20)	0.841
**Employment status**						
Unemployed	220 (9.61)	541 (19.13)	Reference		Reference	
Employed	431 (18.83)	546 (19.31)	0.52 (0.42–0.63)	< 0.001	0.94 (0.70–1.27)	0.698
Self employed	1638 (71.56)	1741 (61.56)	0.43 (0.36–0.51)	< 0.001	1.05 (0.79–1.38)	0.753

Also, 2.74%, 1.45%, 5.58%, 5.05%, and 11.15% of the women tested positive for syphilis, HIV, hepatitis B surface antigen, G6PD deficiency and Hb sickling respectively (Table 2). Only 17 out of 4669 (0.36%) urine samples examined were positive for *Schistosoma haematobium* ova; all positive samples belonging to women from Volta region, (17/3096). No hookworm, Ascaris lumbricoides or Trichuris trichiura ova were detected in all stool samples examined (4843) however, other pathogens like Entamoeba coli, Entamoeba histolytica and Giardia lamblia were detected in only 3.00% of stool samples.

### Factors associated with maternal anaemia at booking ANC visit

Table 4 presents results of unadjusted and adjusted odds ratios obtained from the bivariate and multivariate models respectively. In the final model, study region, age, malaria parasite infection, gestational age at booking and wealth status were significantly associated with maternal anaemia at booking. The odds of maternal anaemia were significantly higher in women enrolled in the Volta region (Adjusted Odds Ratio (AOR) = 3.21, *p* < 0.001) compared to those enrolled in the Ashanti region; women with malaria parasite infection (AOR = 2.09, *p* < 0.001) compared to those with no malaria parasite infection and women reporting later than the first trimester for antenatal booking (AOR = 1.86; *p* < 0.001 for 2nd trimester and AOR = 2.39; *p* < 0.001 for third trimester) compared to those reporting in the first trimester. However, the odds of maternal anaemia were significantly reduced in older women (AOR = 0.57; *p* < 0.001 for women 25–34 years and AOR = 0.66; *p* = 0.008 for women ≥ 35 years ) compared to women < 25 years and women of increasing wealth index (AOR = 0.71; *p* = 0.009, AOR = 0.66; *p* = 0.006 and AOR = 0.44; *p* < 0.001 for middle, upper middle and upper quintiles respectively) compared to women in the lower quintile. *S. haematobium* infection, gravidity, educational level, employment status and having slept under an ITN the night before booking had statistically significant associations with anaemia at the bivariate analyses level, but their significance was lost in the final model. ITN ownership and reporting at least one symptom at booking had no significant associations with anaemia at both the bivariate and multivariate levels of analyses.

### Factors associated with malaria parasite infection at booking ANC visit

Study region, anaemia, gravidity, reporting a clinical symptom, and lower middle wealth status were significantly associated with malaria parasite infection at booking at the multivariate stage of analyses (Table 3). Women in the Volta region had significantly lower odds (AOR = 0.14; *p* < 0.0001) of having malaria parasite infection at booking compared to those in the Ashanti region. Women who were anaemic and who reported clinical symptoms had significantly higher odds of malaria parasite infection (AOR = 1.83; *p* = 0.001 and AOR = 1.82; *p* < 0.0001 respectively) compared to non-anaemic women and those who did not report any clinical symptom. Although women who booked later than the first trimester had two times the odds of having malaria parasite infection, compared to those who booked ANC in the first trimester, at the bivariate stage of analysis (Unadjusted Odds Ratio (UOR) = 1.98, *p* < 0.0001 and UOR = 2.15, *p* < 0.0001 for 2nd and 3rd trimester respectively), this did not remain significant in the final model. Similarly, age ≥ 25–34 years (UOR = 0.45; *p* < 0.001) and age ≥ 35 years (UOR = 0.47; *p* < 0.001) compared to age < 25 years and primary (UOR = 0.57; *p* = 0.031) and tertiary (UOR = 0.25; *p* < 0.001) educational level compared to no formal education, although significantly associated with malaria parasite infection in the bivariate analysis, lost their significance in the final model. The odds of malaria parasite infection seemed to be decreasing with increasing wealth status at the unadjusted analyses stage, although not statistically significant, but only the lower middle quintile wealth index compared to the lower wealth index, showed statistically significant association in the final model (AOR = 0.48; *p* = 0.04). Ownership and use of ITNs and employment status were not significantly associated with malaria parasite infection at booking in both the bivariate and multivariate analyses.

## Discussion

This study aimed at assessing the prevalence of malaria and anaemia and associated factors among pregnant women at their first ANC visits in the Ashanti and Volta regions of Ghana. The study has shown a low prevalence of malaria but high anaemia prevalence among the women at their booking ANC visit. The overall malaria parasite infection prevalence was almost 6% while prevalence of anaemia was 55%. Living in the Ashanti region, being anaemic, being primigravid, and reporting at least one clinical symptom increased the odds of malaria parasite infection among the study women. Living in the Volta region, younger maternal age, having malaria parasite infection, booking ANC at later gestational age and lower wealth index increased the odds of maternal anaemia.

The low overall prevalence of malaria parasite infection reflects a general decline in the prevalence of malaria parasite infection reported previously among pregnant women, the general population and children in Ghana [39, 40, 63] and globally [1]. Declining malaria transmission and improvements in uptake of malaria control interventions [36, 40] and improved housing conditions over the past two decades [64] may be responsible for this. Improvement in the general living environment and in housing conditions enhances malaria control efforts by reducing transmission levels [65].

There was a marked difference in malaria parasite prevalence between the study regions; 10% in Ashanti region versus approximately 3% in Volta region. This marked difference may be due to differences in the vegetation and malaria transmission patterns and ITN use. Some parts of the Volta region are coastal savannah areas where transmission is lower compared to the Ashanti region which is predominantly forest vegetation with perennial and moderately intense malaria transmission [54]. The Ghana Demographic and Health survey (GDHS) repeatedly reports higher ITN use in Volta region compared to Ashanti region [66, 67] and in this study too, more women (74%) in the Volta region used ITN compared with those in the Ashanti region (26%). Reversely however, Volta region had GMPD seven times that of the Ashanti region (18226 parasites per microlitre in Volta region versus 982 parasites per microlitre in Ashanti region). Higher parasite densities in the presence of lower prevalence of malaria infection among pregnant women have been reported in previous studies [68, 69] and reasons given for this observed phenomenon include increased ITN coverage [69] and reduced antimalarial antibodies [68]. The higher ITN use in the Volta region may have translated into less exposure to mosquito bites and parasites that, over time, may have led to less persistent naturally acquired immunity among the pregnant women. Similarly, greater exposure to mosquito bites and parasites from lesser ITN use in the Ashanti region may imply a more persistent naturally acquired immunity that will subsequently limit parasite density. Further research into the immune response of pregnant women, especially among those living in areas of low malaria infection prevalence, are highly indicated to understand its implication for severity of malaria disease and associated complications. The relatively much higher malaria parasite densities observed in the pregnant women from the Volta region may be calling for greater attention to be paid in diagnosing and treating malaria infection among these women to avoid complications and eventual death.

Women who reported at least one clinical symptom had about 80% increased odds of malaria parasite infection (Table 3), corroborating study findings from Ghana, Burkina Faso, Asia and South America [70–72]. The symptoms reported in this study by most of the women, including headache, lower abdominal pain, loss of appetite and dizziness, were similar to those reported in earlier studies [70–72]. Although MiP in stable transmission settings has typically been described as asymptomatic [6, 7], paying attention to common complaints by pregnant women during their ANC may assist in identifying those infected by the malaria parasite, especially now that MiP prevalence is reducing [40].

Increasing wealth status seemed to be associated with decreased odds of malaria parasite infection as has been reported elsewhere [51, 73] although only increasing wealth index from lower to middle lower status showed significant protection. Improving wealth index, especially among the lowest quintile of women, may mean that more women are economically empowered to make decisions about their own health and improve health behaviours. This may translate into an increased likelihood of ITN use [74] and living in improved housing conditions and environment with minimal breeding sites for mosquitoes. In Uganda, women living in traditionally constructed houses had a 41% increased risk of malaria parasite infection compared to those in modern houses [75].

Participants’ age did not influence malaria parasite infection among the women in this current study although there is evidence in a recent review that younger aged pregnant women < 30 years were more likely to have malaria parasite infection in Africa [72]. Increasing gravidity however was protective of malaria parasite infection as has been previously reported [40, 51, 72, 76]. This observation may be explained by the suggestion that antimalarial antibodies acquired in a previous pregnancy are most likely maintained to protect subsequent pregnancies [76]. This may also be related to higher ITN use among pregnant women with higher gravidity [77]. However, some studies have reported variable associations between gravidity and ITN use [32, 78]. Surprisingly, ITN use was not related to malaria parasite infection in this study, contrary to earlier studies that showed that ITN use in pregnancy was protective of malaria parasite infection [69, 79, 80]. It is possible that such a relationship was not observed because of the lower prevalence of malaria reported here, a phenomenon that has been observed in a low-prevalent area in Ibadan, Nigeria [81].

Pregnant women with primary and tertiary levels of education had significantly reduced odds of malaria parasite infection compared to women with no formal education, although this effect was lost in the final model. Studies elsewhere have reported similar findings of significantly reduced odds of malaria parasite infection with increasing educational level among pregnant women [22, 44, 51]. Increasing levels of education may translate into better knowledge about malaria prevention among the women which may mean better adoption of malaria prevention methods during pregnancy [74].

More than half of the pregnant women, 55%, were anaemic at booking ANC visit, similar to the national prevalence of 51% reported in the 2022 GDHS. It is most likely the women carried a pre-existing anaemia status which was worsened by the pregnancy state. The GDHS reports that in 2014 and 2022, 42% and 40% of women in reproductive age respectively, were anaemic [66, 67] signifying the existence of pre-pregnancy anaemia states amongst the women. More than half of the women, 53%, reported later than the first trimester for their booking ANC visit. These women would have suffered the increasing demand of iron and folate by the growing foetus culminating in the reported anaemia prevalence. Hence the recommendation for pregnant women to start ANC early by the 12th week [31] to enable early commencement of ANC interventions including IFAS is justified. It is not surprising then that increasing gestational age at ANC booking increased the odds of maternal anaemia, similarly reported in a review [82].

Formal education seems to play a role in pregnant women’s anaemic status. Generally, as the level of education increases, the level of anaemia decreases significantly as reported in a review of studies in sSA and in Tanzania [17, 29]. Similarly in this study (Table 4), and in a study by Dosoo et al., [51] as the level of education of the pregnant women increased, the odds of anaemia amongst them decreased although not statistically significant. Higher levels of education may facilitate better knowledge of the causes and prevention of anaemia among the women and thus influence positive behaviours towards anaemia prevention during pregnancy.

Having malaria parasite infection at booking ANC increased a woman’s odds of being anaemic, similarly reported in other regions of Ghana [47, 51, 83]. Malaria infection causes breakdown of both infected and uninfected red blood cells leading to reduced Hb levels [84] and remains an important determinant of anaemia even among pregnant women with low malaria prevalence as shown in this study. Younger age, less than 25 years, was found to increase the odds of anaemia among the study women similar to observations made in other studies [51, 85]. Nevertheless, the effect of age on maternal anaemia prevalence may be variable [16].

As the women’s wealth index increased, their odds of having anaemia reduced significantly (Table 4), similar to reports that economic status influences maternal anaemia [16, 86]. Economic power may translate into more nutritionally adequate diets, less exposure to mosquito bites through improved housing [73] and better health care seeking behaviour, all of which may contribute to reducing anaemia. The influence of wealth could be considered a more significant risk factor for maternal anaemia and may explain the higher prevalence of maternal anaemia among the study women in the Volta Region regardless of the lower malaria parasite prevalence. As of 2020, Volta Region was the fourth poorest region in the country while Ashanti Region had one of the lowest poverty rates [87]. Also, sub-microscopic malaria parasitaemia is found to be predominant in low malaria transmission areas [88] and is associated with maternal anaemia, LBW and other adverse pregnancy outcomes [68]. This could also help explain the higher anaemia prevalence in the Volta region, but sub-microscopic malaria parasitaemia was not studied in this research. There is evidence of higher malaria parasite infection prevalence in the Volta, Greater Accra and Central regions of the country using the polymerase chain reaction (PCR) techniques for diagnosis [22, 85, 89]. In the Central Tongu district of Volta region, Frempong et al. reported a prevalence of 24% as against 8% by microscopy in pregnant women at booking ANC [85]. Quakyi et al. reported an average prevalence 43% using ultrasensitive PCR techniques as against an average prevalence of 4% using microscopy among pregnant women at booking ANC in the Greater Accra region [89]. Similarly, a prevalence of 44% in the dry season and 47% in the rainy season has been reported by Anabire et al. in the Central region using PCR techniques [22]. PCR techniques are more expensive and laborious to conduct, and are not usually employed at the clinic level, but their results give indication of the existence of high levels of sub-microscopic malaria parasite infections in pregnant women which may be contributing to the persistence of anaemia in pregnancy. Thus, studies to understand the role of sub-microscopic malaria parasite infections on anaemia in pregnancy are recommended.

Urinary schistosomiasis, known to be associated with maternal anaemia [25] seemed to be so but only at the bivariate analyses stage of this study (*p* = 0.045). This observed association, despite the overall very low infection prevalence of < 1% may signal some importance of urinary schistosomiasis to maternal anaemia, especially among women living along the Volta Lake. Only study participants enrolled from Volta region had urinary schistosomiasis, possibly hailing from the South Tongu district which is found along the Volta Lake. Frempong et al. reported relatively higher prevalence of 3% and 4% among pregnant women attending ANC in 2 health facilities closer to the Volta Lake in CTD [85] but again, no association with anaemia was found. Further studies using more sensitive tests including serological and immunological tests may be needed to better estimate urinary schistosomiasis prevalence and their effect on maternal anaemia in the Volta Region especially.

### Strengths and limitations

The selection of the study sites was purposefully done and thus the sites may not be representative of the two regions studied which is a limitation to this study. However, the districts selected in the two regions span across rural, semi-urban to urban areas ensuring a fair representation of women across all socio-demographic and economic divides in the study population. This reflected in the results of the study where 20% of the women belonged to each quintile of the wealth index (Table 1). Again, although the study participants were consecutively sampled at the selected ANC clinics with the potential for selection bias, the large number of study participants recruited reduces the likelihood of this sampling error that could have been introduced into the study.

The nutritional status of the pregnant women was not assessed in this study and is a limitation as nutrition is a well-known risk factor for anaemia [28, 29]. However, anthropometric measurements, dietary assessments, haematological indices and biochemical tests for key micronutrient deficiencies related to anaemia among a cross-section of women in this study will be reported in another study later.

This study’s strength lies in reporting on a large number of pregnant women that contributes to increased validity of the study findings. Studying women in multiple districts across two regions also significantly improves the external validity of findings.

## Conclusions

The overall malaria parasitaemia prevalence was low, supporting Ghana’s move towards malaria elimination. However, malaria infection remains an important determinant of maternal anaemia despite its reported low prevalence. Conversely, maternal anaemia prevalence was high and of serious public health importance with more than half the study participants being anaemic.

Efforts aimed at eliminating malaria and controlling anaemia in pregnancy should be strengthened especially among young, first-time pregnant women and should be targeted sub-nationally at the regional and preferably, district levels to improve upon the overall health of pregnant women. Early ANC visits in the first trimester of pregnancy need to be highly encouraged. This study also highlights the importance of socio-economic status of pregnant women as a critical risk factor of anaemia in pregnancy. Particular attention needs to be paid to it in efforts to control anaemia in pregnancy.

## Electronic supplementary material

Below is the link to the electronic supplementary material.

Supplementary Material 1

## Data Availability

The data that support the findings of this study are not openly available due to reasons of on-going data analysis for the main study and are available from the corresponding author upon reasonable request. Data are currently located in controlled access data storage at the University of Health and Allied Sciences, Ghana.

## Abbreviations

AiP: Anaemia in Pregnancy
ANC: Antenatal care
AOR: Adjusted Odds Ratio
AZD: Agortime-Ziope District
CTD: Central Tongu District
DHIMS: District Health Information Management System
EJM: Ejisu-Juaben Municipality
EM: Ejisu Municipality
FBC: Full Blood Count
G6PD: Glucose-6-Phosphate Dehydrogenase
GMPD: Geometric Mean Parasite Density
Hb: Haemoglobin concentration
HIV: Human Immuno Deficiency Virus
IFAS: Iron and Folic Acid Supplementation
IPTp: Intermittent Preventive Treatment of Malaria in Pregnancy
ITN: Insecticide Treated Net
JM: Juaben Municipality
KM: Kumasi Metropolis
LBW: Low birth weight
MCHRB: Maternal and Child health Record Book
MiP: Malaria in pregnancy
PCR: Polymerase Chain Reaction
SED: Sekyere East District
sSA: sub-Saharan Africa
STD: South Tongu District
WHO: World Health Organisation

## References

[1] WHO. World malaria report 2019. Geneva: World Health Organisation; 2019.

[2] WHO, The Global Health Observatory. Anaemia in women and Children. WHO Global Anaemia Estimates, 2021. https://www.who.int/data/gho/data/themes/topics/anaemia_in_women_and_children. Accessed 15 February 2025.

[3] Bauserman M, Conroy AL, North K, Patterson J, Bose C, Meshnick S. An overview of malaria in pregnancy. Semin Perinatol. 2019;43(5):282–90. 30979598 10.1053/j.semperi.2019.03.018 PMC7895297

[4] Rahman MM, Abe SK, Rahman MS, Kanda M, Narita S, Bilano V, Ota E, Gilmour S, Shibuya K. Maternal anemia and risk of adverse birth and health outcomes in low- and middle-income countries: systematic review and meta-analysis. Am J Clin Nutr. 2016;103(2):495–504. 26739036 10.3945/ajcn.115.107896

[5] WHO. A strategic framework for malaria prevention and control during pregnancy in the African region. Brazzaville: WHO Regional Office for Africa; 2004. AFR/MAL/04/1-AFR/MAL//1.

[6] Desai M, ter Kuile FO, Nosten F, McGready R, Asamoa K, Brabin B, et al. Epidemiology and burden of malaria in pregnancy. Lancet Infect Dis. 2007;7:93–104. 17251080 10.1016/S1473-3099(07)70021-X

[7] Takem EN, D’Alessandro U. Malaria in pregnancy. Mediterr J Hematol Infect Dis. 2013;5(1):e2013010. 23350023 10.4084/MJHID.2013.010 PMC3552837

[8] Nair M, Choudhury MK, Choudhury SS, Kakoty SD, Sarma UC, Webster P, Knight M. Association between maternal anaemia and pregnancy outcomes: a cohort study in Assam, India. BMJ Glob Health. 2016;1(1):e000026. 28588921 10.1136/bmjgh-2015-000026 PMC5321311

[9] Daru J, Zamora J, Fernandez-Felix BM, Vogel J, Oladapo OT, Morisaki N, Tuncalp O, Torloni MR, Mittal S, Jayaratne K, Lumbiganon P, Togoobaatar G, Thangaratinam S, Khan KS. Risk of maternal mortality in women with severe anaemia during pregnancy and post partum: a multilevel analysis. Lancet Glob Health. 2018;6(5):e548–54. 29571592 10.1016/S2214-109X(18)30078-0

[10] Smith C, Teng F, Branch E, Chu S, Joseph K. Maternal and perinatal morbidity and mortality associated with anemia in pregnancy. Obstet Gynecol. 2019;134(6):1234. 31764734 10.1097/AOG.0000000000003557 PMC6882541

[11] WHO. World malaria report 2023. Geneva: World Health Organization; 2023.

[12] WHO. World malaria report 2022. Geneva: World Health Organization; 2022.

[13] Yimam Y, Nateghpour M, Mohebali M, Abbaszadeh Afshar MJ. A systematic review and meta-analysis of asymptomatic malaria infection in pregnant women in Sub-Saharan Africa: A challenge for malaria elimination efforts. PLoS ONE. 2021;16(4):e0248245. 33793584 10.1371/journal.pone.0248245 PMC8016273

[14] Engdaw GT, Tesfaye AH, Feleke M, Negash A, Yeshiwas A, Addis W, Angaw DA, Engidaw MT. Effect of antenatal care on low birth weight: a systematic review and meta-analysis in Africa, 2022. Front Public Health. 2023;11:1158809. 37441651 10.3389/fpubh.2023.1158809 PMC10335749

[15] Tessema ZT, Tamirat KS, Teshale AB, Tesema GA. Prevalence of low birth weight and its associated factor at birth in Sub-Saharan Africa: A generalized linear mixed model. PLoS ONE. 2021;16(3):e0248417. 33705473 10.1371/journal.pone.0248417 PMC7951905

[16] Karami M, Chaleshgar M, Salari N, Akbari H, Mohammadi M. Global prevalence of Anemia in pregnant women: A comprehensive systematic review and Meta-Analysis. Matern Child Health J. 2022;26(7):1473–87. 35608810 10.1007/s10995-022-03450-1

[17] Nyarko SH, Boateng ENK, Dickson KS, Adzrago D, Addo IY, Acquah E, Ayebeng C. Geospatial disparities and predictors of anaemia among pregnant women in Sub-Saharan Africa. BMC Pregnancy Childbirth. 2023;23(1):743. 37864203 10.1186/s12884-023-06008-3 PMC10588187

[18] Rouamba T, Samadoulougou S, Ouedraogo M, Hien H, Tinto H, Kirakoya-Samadoulougou F. Asymptomatic malaria and anaemia among pregnant women during high and low malaria transmission seasons in Burkina Faso: household-based cross-sectional surveys in Burkina Faso, 2013 and 2017. Malar J. 2021;20(1):211. 33933072 10.1186/s12936-021-03703-4 PMC8088076

[19] Mlugu EM, Minzi O, Kamuhabwa AAR, Aklillu E. Prevalence and correlates of asymptomatic malaria and Anemia on first antenatal care visit among pregnant women in Southeast, Tanzania. Int J Environ Res Public Health. 2020;17(9):3123. 10.3390/ijerph17093123 PMC7246851 32365839

[20] Eisele TP, Larsen DA, Anglewicz PA, Keating J, Yukich J, Bennett A, Hutchinson P, Steketee RW. Malaria prevention in pregnancy, birthweight, and neonatal mortality: a meta-analysis of 32 National cross-sectional datasets in Africa. Lancet Infect Dis. 2012;12(12):942–9. 22995852 10.1016/S1473-3099(12)70222-0

[21] White NJ, Pukrittayakamee S, Hien TT, Faiz MA, Mokuolu OA, Dondorp AM. Malar Lancet. 2014;383(9918):723–35. 10.1016/S0140-6736(13)60024-0 23953767

[22] Anabire NG, Aculley B, Pobee A, Kyei-Baafour E, Awandare GA, Del Pilar Quintana M, Hviid L, Ofori MF. High burden of asymptomatic malaria and anaemia despite high adherence to malaria control measures: a cross-sectional study among pregnant women across two seasons in a malaria-endemic setting in Ghana. Infection. 2023;51(6):1717–29. 37300587 10.1007/s15010-023-02058-z

[23] Asundep NN, Jolly PE, Carson AP, Turpin CA, Zhang K, Wilson NO, Stiles JK, Tameru B. Effect of malaria and geohelminth infection on birth outcomes in Kumasi, Ghana. Int J Trop Dis Health. 2014;4(5):582–94. 25414840 10.9734/IJTDH/2014/7573 PMC4235765

[24] Yatich NJ, Jolly PE, Funkhouser E, Agbenyega T, Rayner JC, Ehiri JE, Turpin A, Stiles JK, Ellis WO, Jiang Y, Williams JH. The effect of malaria and intestinal helminth coinfection on birth outcomes in Kumasi, Ghana. Am J Trop Med Hyg. 2010;82(1):28–34. 20064991 10.4269/ajtmh.2010.09-0165 PMC2803505

[25] Adam I, Al-Wutayd NAAL, Khamis O. Prevalence of schistosomiasis and its association with anemia among pregnant women: a systematic review and meta-analysis. Parasit Vectors. 2021;14(1):133. 33653391 10.1186/s13071-021-04642-4 PMC7923606

[26] Orish VN, Onyeabor OS, Boampong JN, Acquah S, Sanyaolu AO, Iriemenam NC. The effects of malaria and HIV co-infection on hemoglobin levels among pregnant women in Sekondi-Takoradi, Ghana. Int J Gynaecol Obstet. 2013;120(3):236–9. 23219288 10.1016/j.ijgo.2012.09.021

[27] Ezechi O, Odberg Petterson K, Byamugisha J. HIV/AIDS, tuberculosis, and malaria in pregnancy. J Pregnancy. 2012;2012:140826. 22593828 10.1155/2012/140826 PMC3346987

[28] Nonterah EA, Adomolga E, Yidana A, Kagura J, Agorinya I, Ayamba EY, Atindama S, Kaburise MB, Alhassan M. Descriptive epidemiology of anaemia among pregnant women initiating antenatal care in rural Northern Ghana. Afr J Prim Health Care Fam Med. 2019;11(1):e1–7. 31038334 10.4102/phcfm.v11i1.1892 PMC6489153

[29] Sunguya BF, Ge Y, Mlunde L, Mpembeni R, Leyna G, Huang J. High burden of anemia among pregnant women in Tanzania: a call to address its determinants. Nutr J. 2021;20(1):65. 34238307 10.1186/s12937-021-00726-0 PMC8268339

[30] Desta M, Akibu M, Tadese M, Tesfaye M. Dietary diversity and associated factors among pregnant women attending antenatal clinic in Shashemane, oromia, central Ethiopia: A Cross-Sectional study. J Nutr Metab. 2019;2019:3916864. 30993019 10.1155/2019/3916864 PMC6434279

[31] WHO recommendations on antenatal care for a positive pregnancy experience. In WHO recommendations on antenatal care for a positive pregnancy experience. 2016: 152–152. 28079998

[32] Dun-Dery F, Meissner P, Beiersmann C, Kuunibe N, Winkler V, Albrecht J, Muller O. Uptake challenges of intermittent preventive malaria therapy among pregnant women and their health care providers in the upper West region of Ghana: A mixed-methods study. Parasite Epidemiol Control. 2021;15:e00222. 34632123 10.1016/j.parepi.2021.e00222 PMC8488310

[33] Oppong FB, Gyaase S, Zandoh C, Nettey OEA, Amenga-Etego S, Anane EA, Adda R, Dosoo DK, Owusu-Agyei S, Asante KP. Intermittent preventive treatment of pregnant women in Kintampo area of Ghana with sulphadoxine-pyrimethamine (SP): trends spanning 2011 and 2015. BMJ Open. 2019;9(6):e027946. 31230017 10.1136/bmjopen-2018-027946 PMC6596987

[34] Amoakoh-Coleman M, Arhinful DK, Klipstein-Grobusch K, Ansah EK, Koram KA. Coverage of intermittent preventive treatment of malaria in pregnancy (IPTp) influences delivery outcomes among women with obstetric referrals at the district level in Ghana. Malar J. 2020;19(1):1–13. 32580717 10.1186/s12936-020-03288-4 PMC7315483

[35] Menendez C, Ferenchick E, Roman E, Bardaji A, Mangiaterra V. Malaria in pregnancy: challenges for control and the need for urgent action. Lancet Glob Health. 2015;3(8):e433–4. 26187483 10.1016/S2214-109X(15)00041-8

[36] Ampofo GD, Osarfo J, Aberese-Ako M, Asem L, Komey MN, Mohammed W, Ofosu AA, Tagbor H. Malaria in pregnancy control and pregnancy outcomes: a decade’s overview using Ghana’s DHIMS II data. Malar J. 2022;21(1):303. 36303165 10.1186/s12936-022-04331-2 PMC9615308

[37] GSS NMIMR, ORC M. Ghana Demographic and Health Survey 2003. Calverton, Maryland: GSS, NMIMR, and, Macro ORC. 2004.

[38] GSS GHS. ICFMacro. Ghana demographic and health survey 2008. Key findings. Maryland, USA: GSS,GHS and ICF Macro.: Calverton; 2009.

[39] GSS ICF. Ghana malaria Indicator survey 2019. Accra, Ghana, and Rockville. Maryland, USA: GSS and ICF; 2020.

[40] Osarfo J, Ampofo GD, Tagbor H. Trends of malaria infection in pregnancy in Ghana over the past two decades: a review. Malar J. 2022;21(1):3. 34983534 10.1186/s12936-021-04031-3 PMC8725495

[41] Mockenhaupt FP, Rong B, Till H, Eggelte TA, Beck S, Gyasi-Sarpong C, Thompson WN, Bienzle U. Submicroscopic plasmodium falciparum infections in pregnancy in Ghana. Trop Med Int Health. 2000;5(3):167–73. 10747278 10.1046/j.1365-3156.2000.00532.x

[42] Browne ENL, Maude GH, Binka FN. The impact of insecticide-treated bednets on malaria and anaemia in pregnancy in Kassena‐Nankana district, Ghana: a randomized controlled trial. Tropical Med Int Health. 2001;6(9):667–76. 10.1046/j.1365-3156.2001.00759.x 11555433

[43] Ampofo GD, Tagbor H, Bates I. Effectiveness of pregnant women’s active participation in their antenatal care for the control of malaria and anaemia in pregnancy in Ghana: a cluster randomized controlled trial. Malar J. 2018;17(1):238. 29921302 10.1186/s12936-018-2387-1 PMC6009977

[44] Anabire NG, Aryee PA, Abdul-Karim A, Abdulai IB, Quaye O, Awandare GA, Helegbe GK. Prevalence of malaria and hepatitis B among pregnant women in Northern Ghana: comparing RDTs with PCR. PLoS ONE. 2019;14(2):e0210365. 30726218 10.1371/journal.pone.0210365 PMC6364880

[45] Agyeman YN, Newton SK, Annor RB, Owusu-Dabo E. The effectiveness of the revised intermittent preventive treatment with sulphadoxine pyrimethamine (IPTp-SP) in the prevention of malaria among pregnant women in Northern Ghana. J Trop Med. 2020;2020:2325304. 33299426 10.1155/2020/2325304 PMC7704196

[46] Ofori M, Ansah E, Agyepong I, Ofori-Adjei D, Hviid L, Akanmori B. Pregnancy-associated malaria in a rural community of Ghana. Ghana Med J. 2009;43(1):13–8. 19652749 PMC2709171

[47] Fondjo LA, Addai-Mensah O, Annani-Akollor ME, Quarshie JT, Boateng AA, Assafuah SE, Owiredu EW. A multicenter study of the prevalence and risk factors of malaria and anemia among pregnant women at first antenatal care visit in Ghana. PLoS ONE. 2020;15(8):e0238077. 32822409 10.1371/journal.pone.0238077 PMC7444479

[48] Tay SCK, Nani EA, Walana W. Parasitic infections and maternal anaemia among expectant mothers in the Dangme East district of Ghana. BMC Res Notes. 2017;10(1):1–9. 28057071 10.1186/s13104-016-2327-5 PMC5217638

[49] Mocking M, Savitri AI, Uiterwaal C, Amelia D, Antwi E, Baharuddin M, Grobbee DE, Klipstein-Grobusch K, Browne JL. Does body mass index early in pregnancy influence the risk of maternal anaemia? An observational study in Indonesian and Ghanaian women. BMC Public Health. 2018;18(1):873. 30005609 10.1186/s12889-018-5704-2 PMC6045841

[50] Tibambuya BA, Ganle JK, Ibrahim M. Anaemia at antenatal care initiation and associated factors among pregnant women in West Gonja district, Ghana: a cross-sectional study. Pan Afr Med J. 2019;33:325. 31692871 10.11604/pamj.2019.33.325.17924 PMC6815505

[51] Dosoo DK, Chandramohan D, Atibilla D, Oppong FB, Ankrah L, Kayan K, Agyemang V, Adu-Gyasi D, Twumasi M, Amenga-Etego S, Bruce J, Asante KP, Greenwood B, Owusu-Agyei S. Epidemiology of malaria among pregnant women during their first antenatal clinic visit in the middle belt of Ghana: a cross sectional study. Malar J. 2020;19(1):381. 33097044 10.1186/s12936-020-03457-5 PMC7585211

[52] Ayensu J, Annan R, Lutterodt H, Edusei A, Peng LS. Prevalence of anaemia and low intake of dietary nutrients in pregnant women living in rural and urban areas in the Ashanti region of Ghana. PLoS ONE. 2020;15(1):e0226026. 31978048 10.1371/journal.pone.0226026 PMC6980408

[53] McGuire S. World health organization. Comprehensive implementation plan on maternal, infant, and young child nutrition. Geneva, Switzerland, 2014. Adv Nutr. 2015;6(1):134–5. 25593153 10.3945/an.114.007781 PMC4288273

[54] Awine T, Malm K, Peprah NY, Silal SP. Spatio-temporal heterogeneity of malaria morbidity in Ghana: analysis of routine health facility data. PLoS ONE. 2018;13(1):e0191707. 29377908 10.1371/journal.pone.0191707 PMC5788359

[55] GHS. District health information management system II (DHIMS II). Ghana Health Service. Accra. Accessed 21 June 2019.

[56] Ahadzie-Soglie A, Addai-Mensah O, Abaka-Yawson A, Setroame AM, Kwadzokpui PK. Prevalence and risk factors of malaria and anaemia and the impact of preventive methods among pregnant women: A case study at the Akatsi South district in Ghana. PLoS ONE. 2022;17(7):e0271211. 35877761 10.1371/journal.pone.0271211 PMC9312417

[57] Browner WS, Newman TB. Sample size and power based on the population attributable fraction. Am J Public Health. 1989;79(9):1289–94. 2764209 10.2105/ajph.79.9.1289 PMC1349706

[58] Swysen C, Vekemans J, Bruls M, Oyakhirome S, Drakeley C, Kremsner P, Greenwood B, Ofori-Anyinam O, Okech B, Villafana T, Carter T, Savarese B, Duse A, Reijman A, Ingram C, Frean J, Ogutu B. Clinical trials partnership C. Development of standardized laboratory methods and quality processes for a phase III study of the RTS, S/AS01 candidate malaria vaccine. Malar J. 2011;10:223. 21816032 10.1186/1475-2875-10-223 PMC3220650

[59] WHO. Bench aids for the diagnosis of intestinal parasites. World Health Organization; 2019.

[60] Brummaier T, Archasuksan L, Watthanakulpanich D, Paris DH, Utzinger J, McGready R, Proux S, Nosten F. Improved detection of intestinal helminth infections with a formalin Ethyl-Acetate-Based concentration technique compared to a crude formalin concentration technique. Trop Med Infect Dis. 2021;6(2):51. 10.3390/tropicalmed6020051 PMC8167623 33921041

[61] Vyas S, Kumaranayake L. Constructing socio-economic status indices: how to use principal components analysis. Health Policy Plan. 2006;21(6):459–68. 17030551 10.1093/heapol/czl029

[62] WHO. Haemoglobin concentrations for the diagnosis of anaemia and assessment of severity. Vitamin and Mineral Nutrition Information System. Geneva, World Health Organization, (WHO/NMH/NHD/MNM/11.1) (http://www.who.int/vmnis/indicators/haemoglobin.pdf. 2011. Accessed 20 September 2023.

[63] Aregawi M, Malm KL, Wahjib M, Kofi O, Allotey NK, Yaw PN, Abba-Baffoe W, Segbaya S, Owusu-Antwi F, Kharchi AT, Williams RO, Saalfeld M, Workneh N, Shargie EB, Noor AM, Bart-Plange C. Effect of anti-malarial interventions on trends of malaria cases, hospital admissions and deaths, 2005–2015, Ghana. Malar J. 2017;16(1):177. 28446198 10.1186/s12936-017-1828-6 PMC5406984

[64] GSS. Ghana 2021 population and housing census. Preliminary report. Ghana Statistical Service; 2021.

[65] Carter R, Karunaweera ND. The role of improved housing and living environments in malaria control and elimination. Malar J. 2020;19(1):385. 33129327 10.1186/s12936-020-03450-y PMC7603669

[66] GSS. GHS, international I. Ghana Demographic and Health Survey 2014. Rockville, Maryland, USA: GSS, GHS and ICF International. 2015.

[67] GSS ICF. Ghana demographic and health survey 2022: key indicators report. Accra, Ghana, and Rockville. Maryland, USA: GSS and ICF.; 2023.

[68] Mayor A, Bardaji A, Macete E, Nhampossa T, Fonseca AM, Gonzalez R, Maculuve S, Cistero P, Ruperez M, Campo J, Vala A, Sigauque B, Jimenez A, Machevo S, de la Fuente L, Nhama A, Luis L, Aponte JJ, Acacio S, Nhacolo A, Chitnis C, Dobano C, Sevene E, Alonso PL, Menendez C. Changing trends in P. falciparum burden, immunity, and disease in pregnancy. N Engl J Med. 2015;373(17):1607–17. 26488692 10.1056/NEJMoa1406459

[69] Feng G, Simpson JA, Chaluluka E, Molyneux ME, Rogerson SJ. Decreasing burden of malaria in pregnancy in Malawian women and its relationship to use of intermittent preventive therapy or bed Nets. PLoS ONE. 2010;5(8):e12012. 20700457 10.1371/journal.pone.0012012 PMC2917365

[70] Tagbor H, Bruce J, Browne E, Greenwood B, Chandramohan D. Malaria in pregnancy in an area of stable and intense transmission: is it asymptomatic? Trop Med Int Health. 2008;13(8):1016–21. 18631316 10.1111/j.1365-3156.2008.02111.x

[71] Tahita MC, Tinto H, Menten J, Ouedraogo JB, Guiguemde RT, van Geertruyden JP, Erhart A, D’Alessandro U. Clinical signs and symptoms cannot reliably predict plasmodium falciparum malaria infection in pregnant women living in an area of high seasonal transmission. Malar J. 2013;12:464. 24373481 10.1186/1475-2875-12-464 PMC3877878

[72] van Eijk AM, Stepniewska K, Hill J, Taylor SM, Rogerson SJ, Cottrell G, Chico RM, Gutman JR, Tinto H, Unger HW, Yanow SK, Meshnick SR, Ter Kuile FO, Mayor A. Subpatent malaria in pregnancy G. Prevalence of and risk factors for microscopic and submicroscopic malaria infections in pregnancy: a systematic review and meta-analysis. Lancet Glob Health. 2023;11(7):e1061–74. 37276878 10.1016/S2214-109X(23)00194-8 PMC10880462

[73] Degarege A, Fennie K, Degarege D, Chennupati S, Madhivanan P. Improving socioeconomic status May reduce the burden of malaria in sub saharan Africa: A systematic review and meta-analysis. PLoS ONE. 2019;14(1):e0211205. 30677102 10.1371/journal.pone.0211205 PMC6345497

[74] Tesfaye T, Mengistie Alemu B, Egata G, Bekele H, Taye Merga B, Eshetu B, Balis B. Insecticide-Treated Nets utilization and associated factors among pregnant women in Miesso woreda, Eastern Ethiopia: observational study. Int J Womens Health. 2022;14:445–53. 35392502 10.2147/IJWH.S357942 PMC8979939

[75] Okiring J, Olwoch P, Kakuru A, Okou J, Ochokoru H, Ochieng TA, Kajubi R, Kamya MR, Dorsey G, Tusting LS. Household and maternal risk factors for malaria in pregnancy in a highly endemic area of Uganda: a prospective cohort study. Malar J. 2019;18(1):144. 31014336 10.1186/s12936-019-2779-x PMC6480498

[76] Fowkes FJ, McGready R, Cross NJ, Hommel M, Simpson JA, Elliott SR, Richards JS, Lackovic K, Viladpai-Nguen J, Narum D, Tsuboi T, Anders RF, Nosten F, Beeson JG. New insights into acquisition, boosting, and longevity of immunity to malaria in pregnant women. J Infect Dis. 2012;206(10):1612–21. 22966126 10.1093/infdis/jis566 PMC3475637

[77] Mwangu LM, Mapuroma R, Ibisomi L. Factors associated with non-use of insecticide-treated bed Nets among pregnant women in Zambia. Malar J. 2022;21(1):290. 36221068 10.1186/s12936-022-04313-4 PMC9555102

[78] Singh M, Brown G, Rogerson SJ. Ownership and use of insecticide-treated Nets during pregnancy in sub-Saharan Africa: a review. Malar J. 2013;12:268. 23914731 10.1186/1475-2875-12-268 PMC3734149

[79] Gamble C, Ekwaru PJ, Garner P, ter Kuile FO. Insecticide-treated Nets for the prevention of malaria in pregnancy: a systematic review of randomised controlled trials. PLoS Med. 2007;4(3):e107. 17388668 10.1371/journal.pmed.0040107 PMC1831739

[80] Fokam EB, Ngimuh L, Anchang-Kimbi JK, Wanji S. Assessment of the usage and effectiveness of intermittent preventive treatment and insecticide-treated Nets on the indicators of malaria among pregnant women attending antenatal care in the Buea health district, Cameroon. Malar J. 2016;15:172. 26987387 10.1186/s12936-016-1228-3 PMC4794838

[81] Bello FA, Ayede AI. Prevalence of malaria parasitaemia and the use of malaria prevention measures in pregnant women in Ibadan, Nigeria. Ann Ib Postgrad Med. 2019;17(2):124–9. 32669988 PMC7358809

[82] Fite MB, Roba KT, Oljira L, Tura AK, Yadeta TA. Compliance with Iron and folic acid supplementation (IFAS) and associated factors among pregnant women in Sub-Saharan Africa: A systematic review and meta-analysis. PLoS ONE. 2021;16(4):e0249789. 33852614 10.1371/journal.pone.0249789 PMC8046188

[83] Anlaakuu P, Anto F. Anaemia in pregnancy and associated factors: a cross sectional study of antenatal attendants at the Sunyani municipal hospital, Ghana. BMC Res Notes. 2017;10(1):402. 28800737 10.1186/s13104-017-2742-2 PMC5553653

[84] White NJ. Anaemia and malaria. Malar J. 2018;17(1):371. 30340592 10.1186/s12936-018-2509-9 PMC6194647

[85] Frempong NA, Ahiabor C, Anyan WK, Mama A, Kusi KA, Ofori MF, Adu B, Debrah AY, Anang AK, Ndam NT, Courtin D. Malaria, urogenital schistosomiasis, and anaemia in pregnant Ghanaian women. J Parasitol Res. 2023;2023:7500676. 37808169 10.1155/2023/7500676 PMC10558271

[86] Samuel S, Darebo T, Desta DT, Mulugeta A. Socio-economic and dietary diversity characteristics are associated with anemia among pregnant women attending antenatal care services in public health centers of Kembata Tembaro zone, Southern Ethiopia. Food Sci Nutr. 2020;8(4):1978–86. 32328264 10.1002/fsn3.1485 PMC7174199

[87] UNDP. New data looking at poverty in different dimensions in Ghana show reduction over time. United Nations Development Programme. 2020. https://www.undp.org/ghana/press-releases/new-data-looking-poverty-different-dimensions-ghana-show-reduction-over-time. Accessed 30 July 2023.

[88] Whittaker C, Slater H, Nash R, Bousema T, Drakeley C, Ghani AC, Okell LC. Global patterns of submicroscopic plasmodium falciparum malaria infection: insights from a systematic review and meta-analysis of population surveys. Lancet Microbe. 2021;2(8):e366–74. 34382027 10.1016/S2666-5247(21)00055-0 PMC8332195

[89] Quakyi I, Tornyigah B, Houze P, Kusi KA, Coleman N, Escriou G, Laar A, Cot M, Fobil J, Asare GQ, Deloron P, Anang AK, Cottrell G, Ofori MF, Ndam NT. High uptake of intermittent preventive treatment of malaria in pregnancy is associated with improved birth weight among pregnant women in Ghana. Sci Rep. 2019;9(1):19034. 31836735 10.1038/s41598-019-55046-5 PMC6911095

