# Supplementary material for: Malaria and anaemia prevalence and associated factors among pregnant women initiating antenatal care in two regions in Ghana: an analytical cross-sectional study
Source: BMC Pregnancy Childbirth. 2025 May 27;25:617. doi: 10.1186/s12884-025-07735-5 (PMC12108015; doi:10.1186/s12884-025-07735-5)
Supplement: Supplementary file 1 — Supplementary Material 1 [file 12884_2025_7735_MOESM1_ESM.docx]

Additional file 1: Table S1: Frequency of symptoms reported by women at ANC booking.

| **Symptoms reported at booking** | **Frequency** | **Percentage (95%CI)** |
| --- | --- | --- |
| Headache | 772 | 22.65 (21.25 - 24.09) |
| Lower abdominal pain | 664 | 19.48 (18.16 - 20.85) |
| Loss of appetite | 357 | 10.47 (9.46 - 11.55) |
| Dizziness | 334 | 9.80 (8.82 - 10.84) |
| Waist pains | 323 | 9.47 (8.51 - 10.51) |
| Vomiting | 288 | 8.45 (7.54 - 9.43) |
| Body pains | 246 | 7.22 (6.37 - 8.14) |
| Easily tired | 216 | 6.34 (5.54 - 7.21) |
| Chills | 82 | 2.41 (1.92 - 2.98) |
| Fever | 76 | 2.23 (1.76 - 2.78) |
| Vaginal discharge | 51 | 1.50 (1.12 - 1.96) |
